# Eureka Moments Shared by Chemists. Hints at Enhancing One’s Own Creativity (and Even One’s Joy)

**DOI:** 10.1021/acscentsci.4c00802

**Published:** 2024-10-31

**Authors:** Jeffrey I. Seeman, Judy I. Wu

**Affiliations:** † University of Richmond, Richmond, Virginia 23173, United States; ‡ University of Houston, Houston, Texas 77204, United States

## Abstract

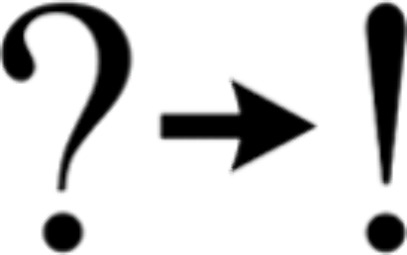

Eureka moments can occur during all steps of discovery. Eighteen chemists and molecular scientists described their Eureka moments herein. Hints at fostering one’s own Eureka moments are provided.

We cannot know what other people’s critical moments have been unless they tell us; they are rare and personal. But as educators we may be able to create more of them.^1^

D. Rae, 2013

Any group of people pinned at its intersection will still burst forth every which way, a tapestry of contradictions, noble and ignoble, wild and banal...humanity is always sprawly, fragmented and irrepressible.^2^

D. Duncan, 2023

It is now clear that one must distinguish between science in the sense of the personal struggle and a different, communal activity, also called ‘science,’ which is its public, institutional aspect.^3^

G. Holton, 1988

## Introduction

Historians of science have rarely studied the “thought processes which lead to scientific discoveries”^3^ and the emotions that transpire from making discoveries.^4−11^ Scientists themselves rarely include such personal accounts in their writings,^12^ given that such human experiences are not considered to be a part of a journal article by editors and reviewers.^13−15^ Nonetheless, this void in knowledge reveals an opportunity to improve one’s effectiveness in the scientific processes and to increase joy derived from doing science. In specific, we were curious about Eureka moments in chemical discovery as well as events leading to the happening of Eureka moments.

During the course of scientific discovery, special emotions can be experienced by the scientists. These feelings are often accompanied by moments of inspiration.^16^ Some scholars refer to these “Eureka moments,” as “aha feelings,” or as “light bulb moments.” These are often transformative^17^ for the chemist and progressive for chemistry.^5,7^ We make the distinction that a Eureka moment is an often experienced part of the process of scientific discovery but does not represent the complete act of a scientific discovery. A Eureka moment can occur in any stage of the discovery process, including but not limited to the following: during inductive, imaginative, creative, and willful thinking;^6^ during a mindful search to solve a particular problem,^4,7^ while performing experiments and assessing anomalies;^4^ when in the midst of performing unrelated tasks; or doing “nothing in particular”; and even when dreaming. Indeed, multiple Eureka moments can occur during any one multifaceted discovery period. As Daniel E. Koshland has said,

“Most important discoveries are usually not solved in one “Eureka” moment....True, there are moments in which a scientist has been mulling over various facts and problems and suddenly puts them all together, but most major discoveries require scientists to make not one but a number of original discoveries and to persist in pursuing them until a discovery is complete.^7^

This article is focused on the human side of chemistry and, in particular, on Eureka moments leading up to chemical discoveries with their corresponding periods of joy in the revelation of chemical insight.^18^ As these deeply personal events are hardly ever described in scientific publications, they are invisible to others. There are very few testaments to Eureka moments in chemistry though there are some in other disciplines.^19^ But we know from our own personal experiences that chemists do have these experiences.

Incorporating these events into the story of chemistry can enhance our understanding of the meaning of life within chemistry.^20^ Furthermore, a knowledge of these Eureka moments can help frame innovations within the broader context of progress in chemistry.^21^ We hope to further identify chemists’ relationships with innovation. It is possible that we can learn from the experiences of others to enhance our own potential for discovery.

We interviewed a diverse cohort of 18 scientists representing varied subdisciplines in chemistry, nationalities, ethnicities, genders and races. Our study was not limited to Eureka experiences of any one type. Rather, we asked our interviewees about moments of enlightenment that evoked feelings of joy or even feelings of enchantment. In each case, we asked the interviewee to identify a Eureka moment they experienced. We emphasized that we were searching for a moment in their professional life in which there was a discontinuity in knowledge, for a moment, just before which to just after which, caused them to see a problem differently or come to a new understanding. For each, we focused on personal experiences. What we did not do was ask the interviewees to identify the most important Eureka moment they ever experienced, just one which they could remember clearly. The vignettes that follow have been edited and condensed from one or sometimes multiple conversations, and all have the approval of the interviewees.

The overall goals of this project were

- To encourage awareness and self-reflection as it relates to discovery;
- To examine diversity and pluralism^22−24^ in creativity and Eureka moments;
- To improve decision making and intuitive judgments in research;
- To enhance the possibility of Eureka moments and productive strategies in research success;
- To place Eureka moments in their proper perspective; and
- To recognize the omnipresence of emotional engagements in research.

## The Stories

Authors’ note: All the interviews in this publication were performed by the authors between December 2023 and May 2024. The subtitles that appear with each vignette represent some combination of definitions of what a Eureka moment is for each interviewee and the different conditions that can clear the way or make space for each Eureka moment. These subtitles are intended to be visionary and pluralistic. All portraits were prepared by J. I. Wu.

### Igor Alabugin: It is connecting the dots that everyone sees, but in different ways

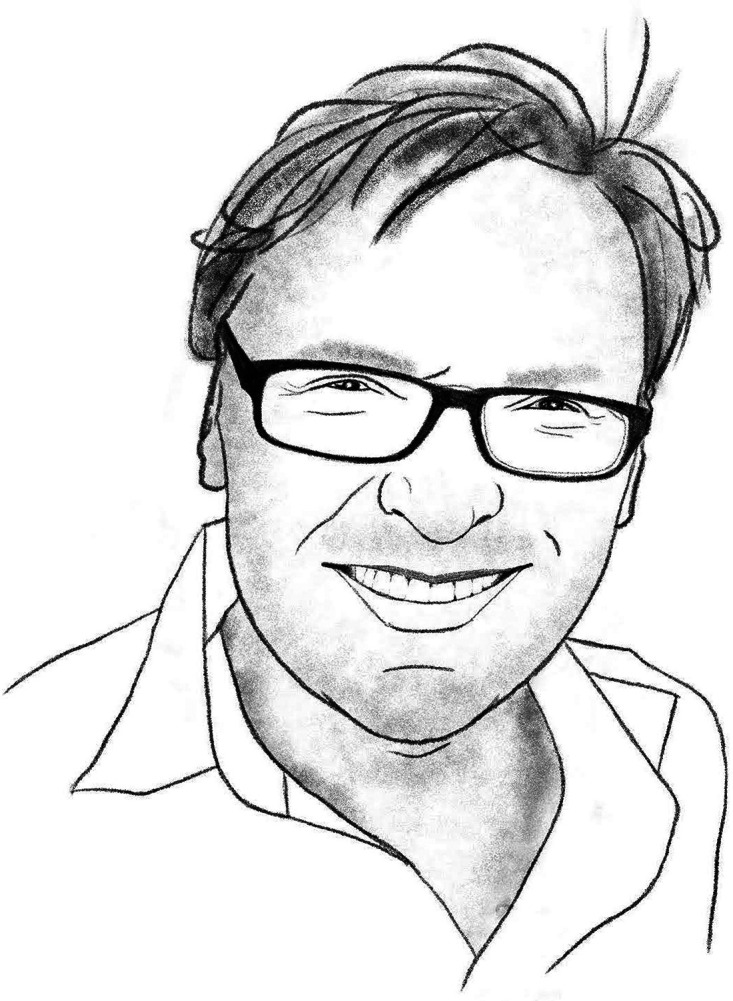



Igor Alabugin was an undergraduate intern during the summer of 1988, working in the R&D laboratory of a chemical plant in Novomoskovsk, Russia, some 230 km south of Moscow and countless thousands of kilometers west of his hometown in Siberia. Part of his assignment in the process development department was the reduction of a black tar that resulted sometimes, not all the time, in large-scale recovery of a certain expensive high-boiling solvent. After four or five years of experiments by the full-time staff, little progress had been made to improve the process. One chemist had found that when potassium permanganate was added to the mixture in this high temperature distillation, tar formation was decreased. According to Alabugin,

“I went to the library where they had *JACS* [*Journal of the American Chemical Society*] and *JOC* [*The Journal of Organic Chemistry*] and began reading through them. I came across a publication that discussed a polymerization that was promoted by low oxidation state transition metals. I wondered, ‘Could that be the answer? That from batch to batch, there could be variable amounts of some transition metal impurities. How could I show that?’

“I rushed back to the laboratory, took a few test tubes, added some metal salts to the reaction mixture, started the reaction and saw a variety of color changes, from light yellow to dark color. Only low oxidation state metals in the reaction were causing decomposition! And an oxidant could convert those impurities to innocuous substances. Equally exciting was testing the new idea of adding a chelating agent that could precipitate the metal impurities and finding that the solution was left with the original pristine color after heating.

“How did I feel? I can still remember the emotions, sitting there in the library. Coming over me was a sense of anticipation along with vibrant elation. I felt like a Christmas present was waiting for me in the laboratory, a new bicycle! I was almost certain I’d find it!”

### William M. Clemons, Jr.: An experimental moment after years of hard work and false trajectories

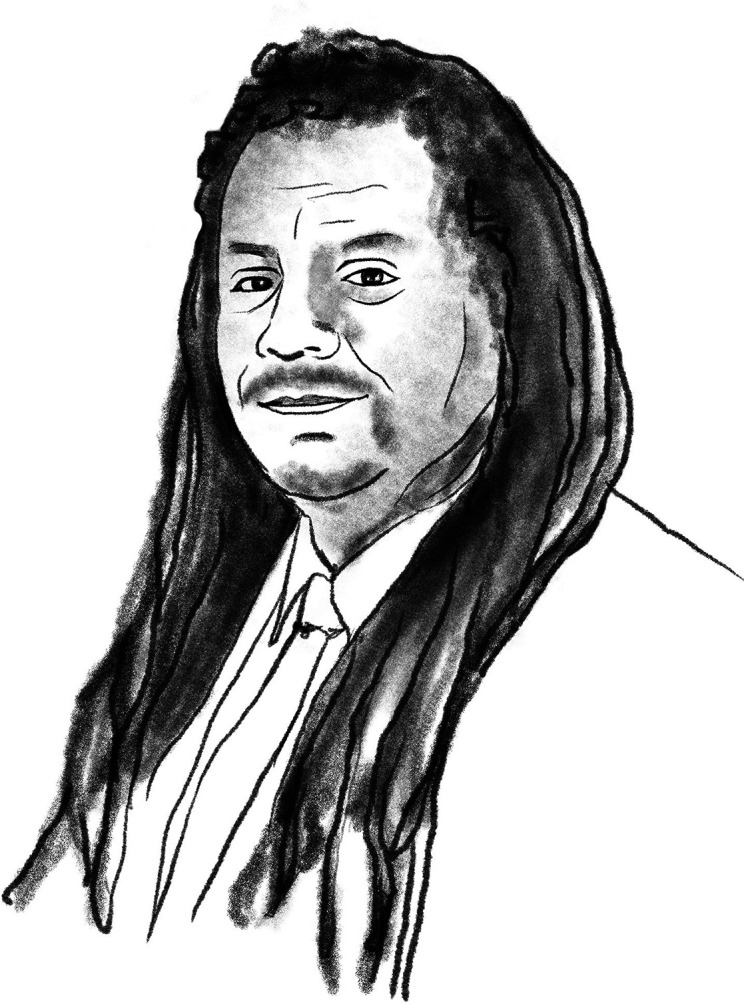



For Bil Clemons, it was more of a mechanical than intellectual Eureka moment. His goal was to understand the structure of a protein-conducting channel in a conserved heterotrimeric membrane protein complex. His experimental task was to obtain crystals of this protein, more formally known as the Sec61 or SecY complex from *Methanococcus jannaschii*, that were suitable for X-ray crystallographic analysis. But not all the crystals he grew were suitable. For each crystal he grew—and growing crystals of proteins is never a trivial experimental endeavor—he obtained X-ray diffraction data. But the real challenge was, and always is, to experimentally obtain phases that will ultimately provide the electron density map calculated from the obtained diffraction pattern. On a Friday evening early in 2003, after two years of painstaking postdoctoral research, the Eureka moment arrived in the form of a graphic on Clemons’ computer monitor: the first useable and useful electron density map for this protein.^25^

“I was ecstatic. I was on Cloud Nine. I was at the top of that mountain. The feeling was almost magical. I can see it all, feel it all, even today.”

Further analysis revealed a remarkable structure in which a cytoplasmic funnel leads into a protein-conducting channel that itself opens into an “hourglass” passageway “having a ring of hydrophobic resides at its construction.”^25^

Clemons later summarized his joys over a broader time-period. He said,

“Structural biology is definitely more like being the first to get to the top of a mountain or discover a new land. There’s lots of new, small Eureka moments in the classic sense.”

### Joe Francisco: Having a special time in the year for innovation

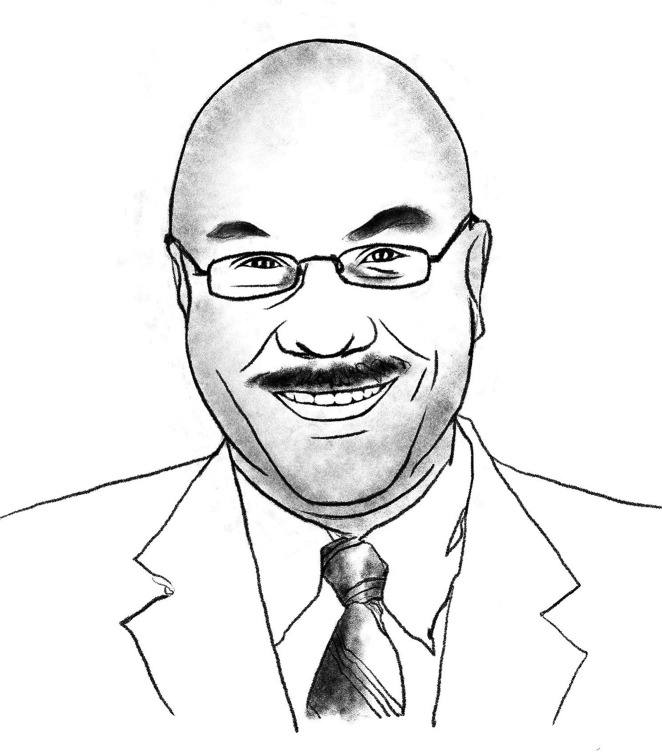

“It’s all about Christmas Eve. I am with my family, a very relaxing time. It’s quiet, the kids are watching TV or are outside, everyone is doing their own thing, emails go dormant, life seems to shut down, everyone retreats to themselves, I’m just chilling out. This is the time for me when ideas germinate, bubble up. I look at what are interesting problems, I prod around and explore. I search for the profound. Some years, I even run some tests or do a few calculations. You ask for a specific example? [Francisco smiles broadly.] Around 1998,^26^ I was thinking about models for atmospheric chemistry and noticed the absence of water in those models. All the laboratory work had been done under pristine yet anhydrous conditions. I realized that models without water rarely reflect what’s going on in the atmosphere. An idea germinated, bubbled up. And that opened up an entire field.^27,28^

“What did I feel? No immediate emotion, just the curiosity to explore.”

When asked if he can dial-up these creative moments at other times, Francisco responded,

“No, I never try to reinvent Christmas Eves at other times of the year. I believe that when one is under pressure to make a big discovery, it cannot happen. Just chilling out and letting things bubble up...”

### Danna Freedman: Gathering, assimilating, then restructuring

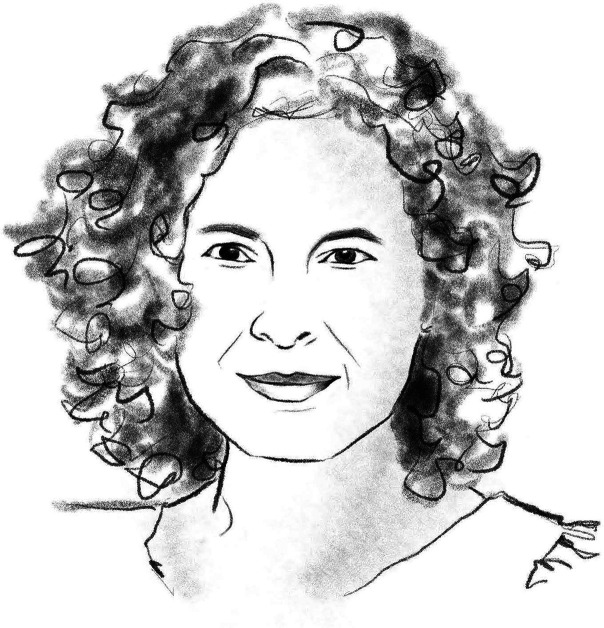



For inorganic chemist-materials scientist Danna Freedman, moments of breakthrough creativity most often occur when she goes for an intentional innovation walk.

“If there’s an idea I want to kick around, if there are ideas in my head and the pieces need to be rearranged, I go for a long walk.

“I was a Postdoc and was writing my research proposals for my independent research career. I was playing around with ideas related to electronic properties of new materials, heavy metals, and magnetic [dipole] moments. I was walking outside and talking on the phone with a friend. Indeed, I was arguing, defending my position in that argument, trying to find my position, jostling ideas around in my head. I suddenly had the realization that I had framed the problem incorrectly. The realization was that a single compound having two atoms, one a source of the spin and the other the course of the angular momentum. A compound containing iron and bismuth.^29^ I then knew I had a novel idea for my research proposal that no one else was going to have. I felt ‘complete’. I had gone from a feeling of “ephemeral” to ‘solid, deep satisfaction.’”

When asked, “Do long walks always lead to creative moments?” Freedman responded,

“One possible conclusion of a walk is the recognition that I don’t always know all the pieces. I need to find more information, but in general, walking, or running helps me think of ideas.”

### Mahesh Hariharan: Across realms into an orthogonal discipline

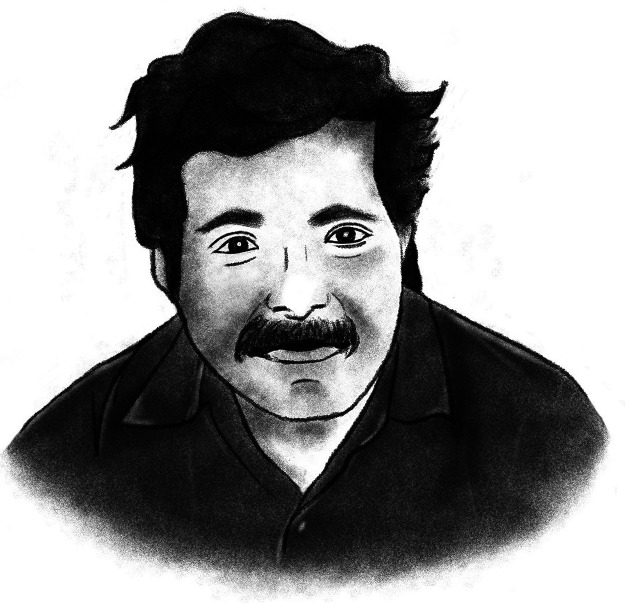



For Mahesh Hariharan it is bringing a good question across realms to a different research area.

“During my postdoc time with Fred Lewis at Northwestern University [2007–2009] working on the dynamics of charge transport in DNA, I thought a lot about why charge transport wasn’t very efficient in DNA.

“You see, nature has chosen to align base-pairs in a twist stacked form by 36°. You cannot have base pairs perfectly stacked on top of each other with a 0° twist angle because there is orbital repulsion. I wondered what would happen if you had a rotational offset of 90°? What are the limits to charge transport?

“These questions followed me into my independent career as I ventured into the field of crystal engineering and chromophore design.”

Mahesh recalls the day his group captured the crystal structure of two orthogonally cross-stacked optical chromophores.

“The moment we got a crystal structure of our first crossed-aggregate,^30^ we knew the importance of it. People knew about how transition dipoles behave in J- and H-aggregates, but transition dipole coupling in crossed-aggregates were never considered. This opened up opportunities for understanding excited-state dynamics!”

Even though the question was clear all along, the route to making an orthogonally cross-stacked dimer was not apparent.

**Figure 1:**
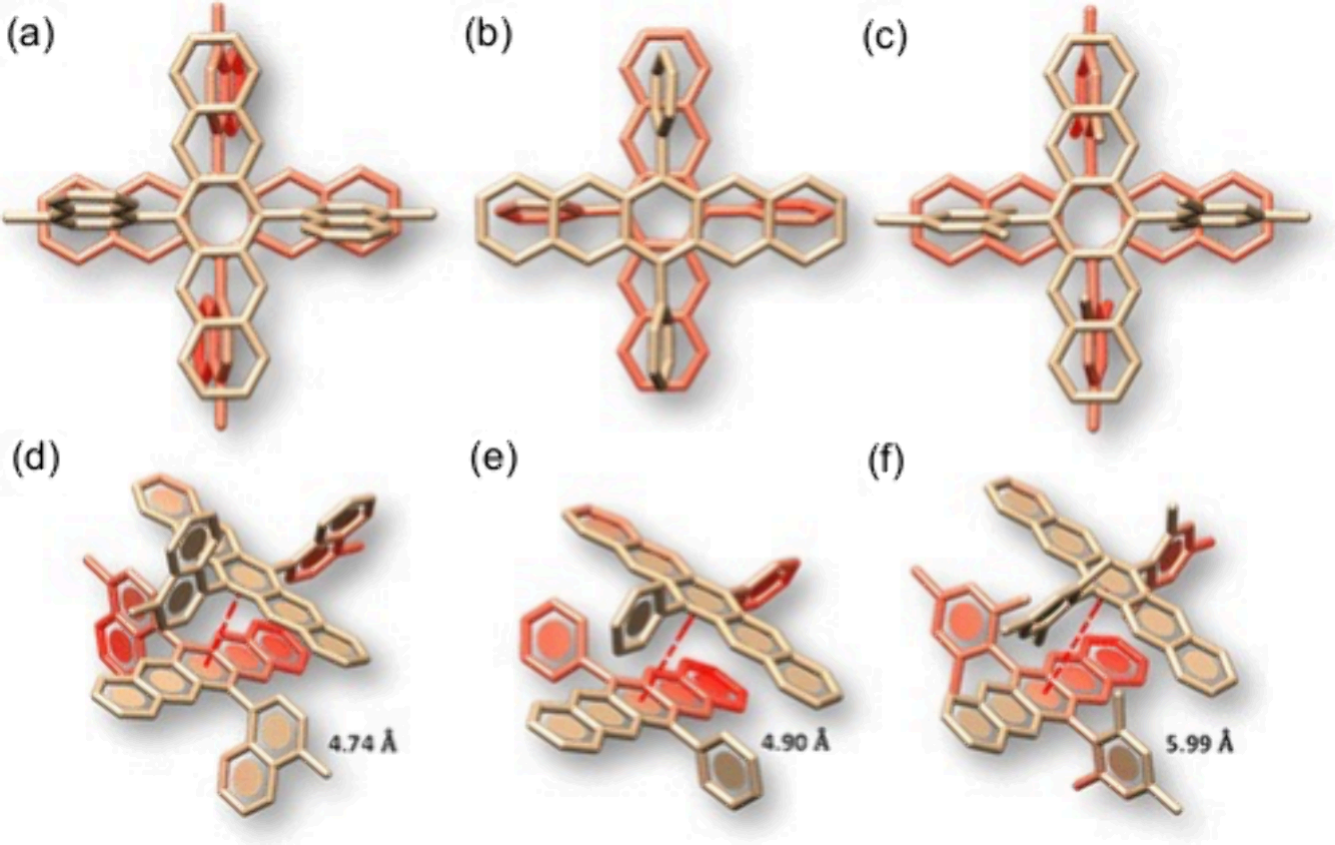
Orthogonally cross-stacked dimers in crystalline (a) 6,13-bis(4-methylnaphthalen-1-yl)pentacene, (b) 6,13-diphenylpentacene, and (c) 6,13-dimesitylpentacene. Schematic representation demonstrating intermolecular distance in (d) N2−P, (e) P2−-P, and (f) M2−P. Reprinted with permission from ref (30). Copyright 2020 American Chemical Society.

“Before we made a crossed-aggregate, we knew from theory that electronic coupling for such structures should be low.^31^ The challenge was how to design it. We started by designing some routine experiments but refined our ideas and learned from our failures until we stumbled upon our first crossed-aggregate dimer [Figure 1].”^30,31^

### Roald Hoffmann: It’s intermittently following an idea and years later, observing and explaining an unusual data point

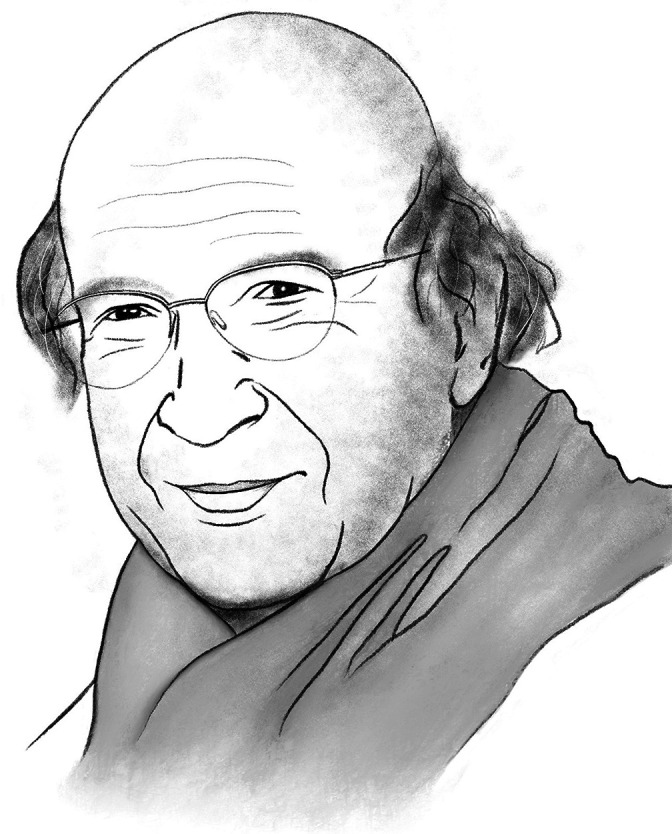



In May 1964, in his second publication featuring his earliest extended Hückel theory (eHT) calculations, Roald Hoffmann reported the delocalization of the lone pair electrons in azines, e.g., in pyridine and pyrazine. He wrote,

“Clearly, the presumed lone pair [in the ring nitrogen] mixes with other σ-orbitals.”^32^

Hoffmann was then in his first months as a Junior Fellow in Harvard’s prestigious Society of Fellows. During the next few years, Hoffmann studied and published (in somewhat chronological order) further eHT calculations on boron and nitrogen compounds, carbocations, and (with R. B. Woodward) on orbital symmetry control of pericyclic reactions.^33−39^ After his move to Cornell in June 1965, Hoffmann’s research was multidirectional, where he published on cumulenes, photochemistry of diazirines and diazomethanes, isomer stability, the spiranes, methylenes and trimethylene, clearly, a wide variety of projects. Hoffmann had one research characteristic that is germane to this discussion. He was persistent in his interests, and he would continue projects, intermittently though regularly, until their completion.

**Figure 2:**
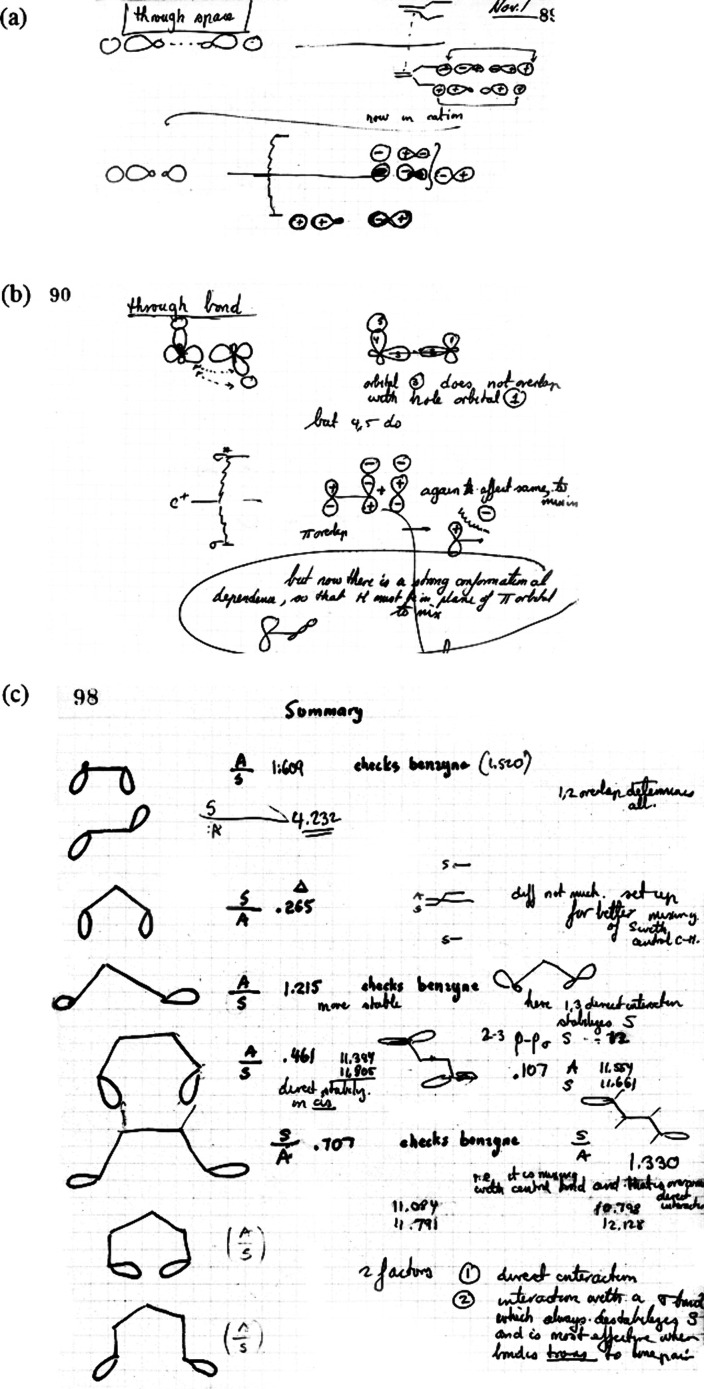
Excerpts from Roald Hoffmann’s *Laboratory Notebook 18*, early November 1965. (a) From page 89, “through space” interactions. (b) From Page 90, “through bond” interactions. (c) From page 98, “Summary” of through bond coupling or delocalization.

In early November 1965, Hoffmann returned to his study of through space and through bond mixing or delocalization. He performed eHT calculations on dehydronaphthalenes, dehydrophenanthrenes, dehydrobiphenylenes, dehydroazulenes, and dehydropolyenes and compared the results with the three dehydrobenzenes (see Figure 2). And in 1968, together with a precocious Cornell undergraduate Warren J. Hehre and Akira Imamura, a postdoctoral student from Kenichi Fukui’s group, Hoffmann reported what he considers to be one of his most stunning accomplishments: “the interaction of orbitals separated by a number of intervening σ-bonds,” i.e., through bond coupling.^40^ That 1968 publication featured advances in theory fueled by more extensive molecular orbital calculations from which Hoffmann et al.

“deduced significant and specific interactions among radical lobes in the same molecule separated by a number of intervening σ-bonds. The interaction is shown to depend only on the orientation of the σ-bonds between the radical lobes and the orientation of the lobes themselves, not on the specific molecule”.^40^

Notable about these discoveries by Hoffmann was the stepwise growth in knowledge separated by several years during which he focused on other projects. In truth, the experience that Hoffmann decided to share for this project does not feel like that single, sudden triumphant moment that was the object of searches. And Hoffmann’s interviewer (JIS) had previously identified with Hoffmann several such Eureka moments that occurred during Hoffmann’s research in early 1965 on what was later termed the Woodward–Hoffmann rules. These Eureka moments have now been documented elsewhere.^41,42^ (And those were, indeed, “sudden triumphant moments.”) But Hoffmann was resistant to include one of those experiences herein. Why? In part because, as he described himself, he was being a “contrarian.” Readers, he felt, would expect his example to come from his orbital symmetry studies, and he wanted to provide a different example. But we, the authors, would counter any judgment that Eureka moments must be of a compressed chronological time. In this example by Hoffmann, he has revealed a Eureka moment that indeed had a sudden triumphant moment, but its course extended over several years.

### Susan Kauzlarich: A mindful choice of research area, a broad research menu, then observing a key discovery and following a new direction

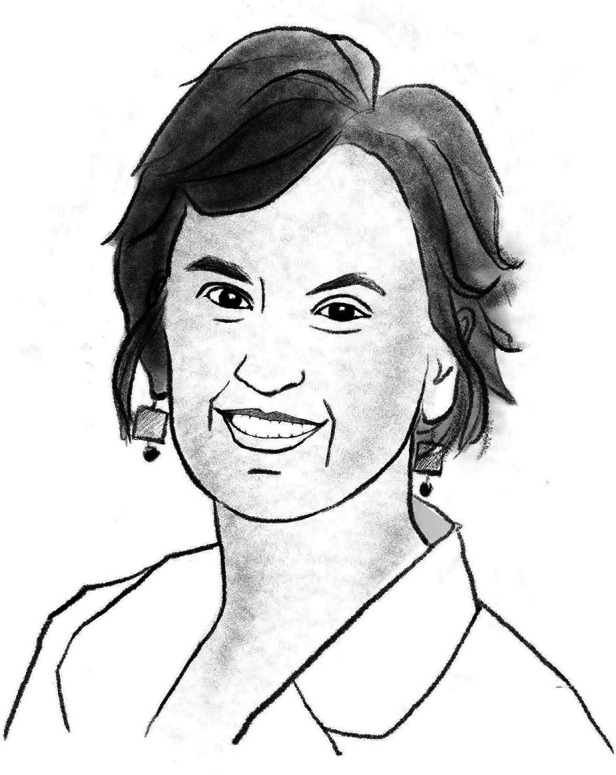



Curiosity drives and directs Susan Kauzlarich. She then looks to connect her research results with real world applications. This tale takes us back to her early days as an assistant professor. She was thinking outside the box, trying to come up with original ideas, bringing together her background in molecular science to solid state science.

“We were making new compounds, going down Group 15 of the periodic table (As, Sb, and Bi), where bonding becomes more delocalized, looking for novel and practical semiconductors. We were investigating their thermoelectric properties, compounds that convert a temperature gradient directly into electricity. Some of our ideas were coalescing. No one hits it big on their first try. But we were so convinced one of our earliest new ternary transition-metal compounds, namely Ca_14_MnBi_11_, was important.^43^ We went on to prepare the rare earth analogs with Eu and Yb and discovered a way to prepare Yb_14_MnSb_11_ in large enough yield to measure its thermoelectric properties. It is still one of the best we’ve ever made.”^44,45^

To be fair, Kauzlarich did not just imagine a compound having that empirical formula. It is isostructural to the Zintl compounds Ca_14_AlSb_11_ and Ca_14_GaAs_11_. As she wrote about Ca_14_MnBi_11_ in 1989,

“The compound is made up of tetrahedra, Bi_3_^7–^ linear chains and isolated Bi^3–^ and Ca^2+^ ions. This is the first reported example of a Mn^III^ tetrahedron and a Bi_3_^7–^ linear anion.^43^

“What was my emotional response to this discovery? I think the idea’s conception took time and energy to convince the reviewing community that there was substance to the concept. However, once I showed that the idea worked and discovered unique properties, I was ecstatic!”

### Robert Langer: From hard work and trial and error to using metaphors as stimuli

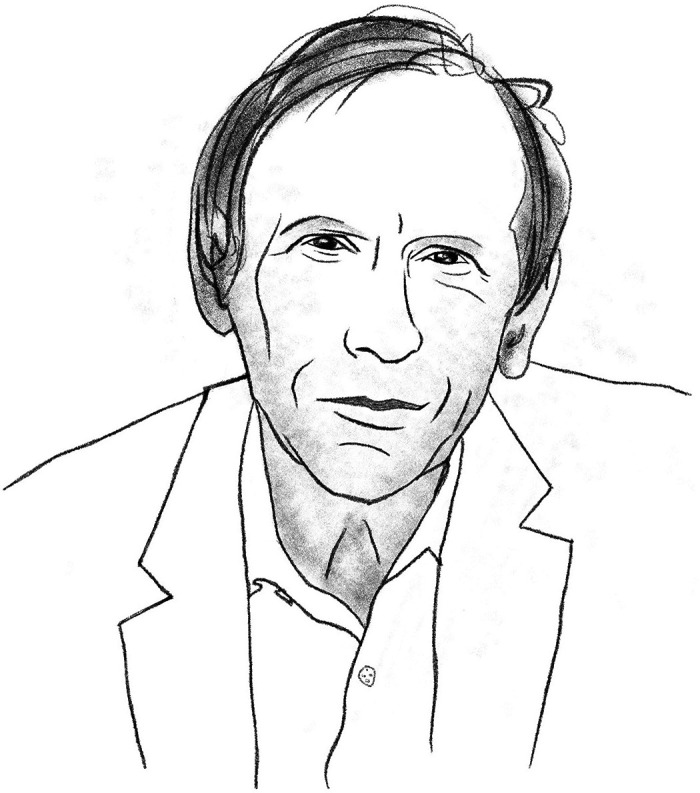



It would certainly be hard to determine if Robert Langer has had more ideas than most people. Nevertheless, he surely has had more “good” ideas than most, if by “good” ideas, one means ideas that have led to applications of relevance to society and their consequential commercial success. Wikipedia lists Langer as being involved in the founding of 33 companies, including Moderna, well-known for its invention and production of one of the two commercial COVID-19 mRNA vaccines.

In a study of innovation in chemistry, it would certainly make sense to ask an acclaimed innovator the source of his ideas. So one of us (JIS) did ask Langer. He immediately and without hesitancy responded with several stories. The first story began in 2001.

“I was on an exercise bicycle at a hotel in Florida where I was soon to give a lecture. I picked up an issue of *Life* magazine and was reading a story of the automobiles of the future. If that car was dented in an accident, you would heat it, and it would snap back into shape. I realized what they were talking about was shape memory. What if we could make materials, such as polymers, that could do this. They would be useful in medicine. For example, you could make surgical sutures that tie knots themselves. This could change minimally invasive surgery. The surgeon could make a hole in a tissue and put something like a string (for example, a surgical suture) through the hole, then when it got to body temperature or by light, it could change shape into whatever you wanted. You could make it tie a surgical knot if you wanted. And we ended up doing that idea. We published it in Science^46^ in 2002 and in Nature^47^ in 2005.”

This invention may lead to next-generation drug-eluting stents and new biomaterial-based drug delivery treatments for cancer, schizophrenia, arthritis, and many other diseases.

The second story is more “mundane from the standpoint of perseverance,” according to Langer but only because it is rather typical of the experiences of a number of scientists. Langer’s goal was to develop sustained release over prolonged periods of time of biochemically active macromolecules from noninflammatory polymeric vehicles which eventually provided the basis for inventions such as drug-eluting stents and nanoparticles that delivered mRNA vaccines.^48,49^ This was in the early 1970s. As Langer reported,

“I tried lots of different things, and they weren’t working at all. Over and over again, maybe I tried 200 things or more....Finally I came up with a design and it [worked].”

Langer was asked, “Was it trial-and-error or did you take knowledge from the past failures, and there was an intelligently designed trajectory?” He responded with a hearty smile and chuckle,

“In this case, some of both. However, sometimes an idea just hits you. It’s almost by accident, like reading the article in *Life* magazine. That was by chance.”

Drug delivery systems based on these research achievements have led to countless products ranging from nanoparticles that deliver COVID-19 mRNA vaccines^50^ to treatments for opioid addiction and arthritis to enabling new treatments in aquaculture as of September 2024. When asked, “Is there any kind of spiritual experience you have when one of these ideas strikes you? Is there some kind of emotional response to a great idea that you have?” Langer responded,

“In the first case, I think you feel happy. Certainly, in the second case I felt happy that I finally got something to work after hundreds of failures.”

For Langer, there is no one path to success. This is another example of the value of pluralism in science.

### David MacMillan: It is the question that matters

MacMillan remembers quite clearly standing for hours and hours every day, working at the glovebox as a postdoc with Dave Evans at Harvard, being “stuck in it” doing research on air and water-sensitive metal-based catalysts. As MacMillan recalled,
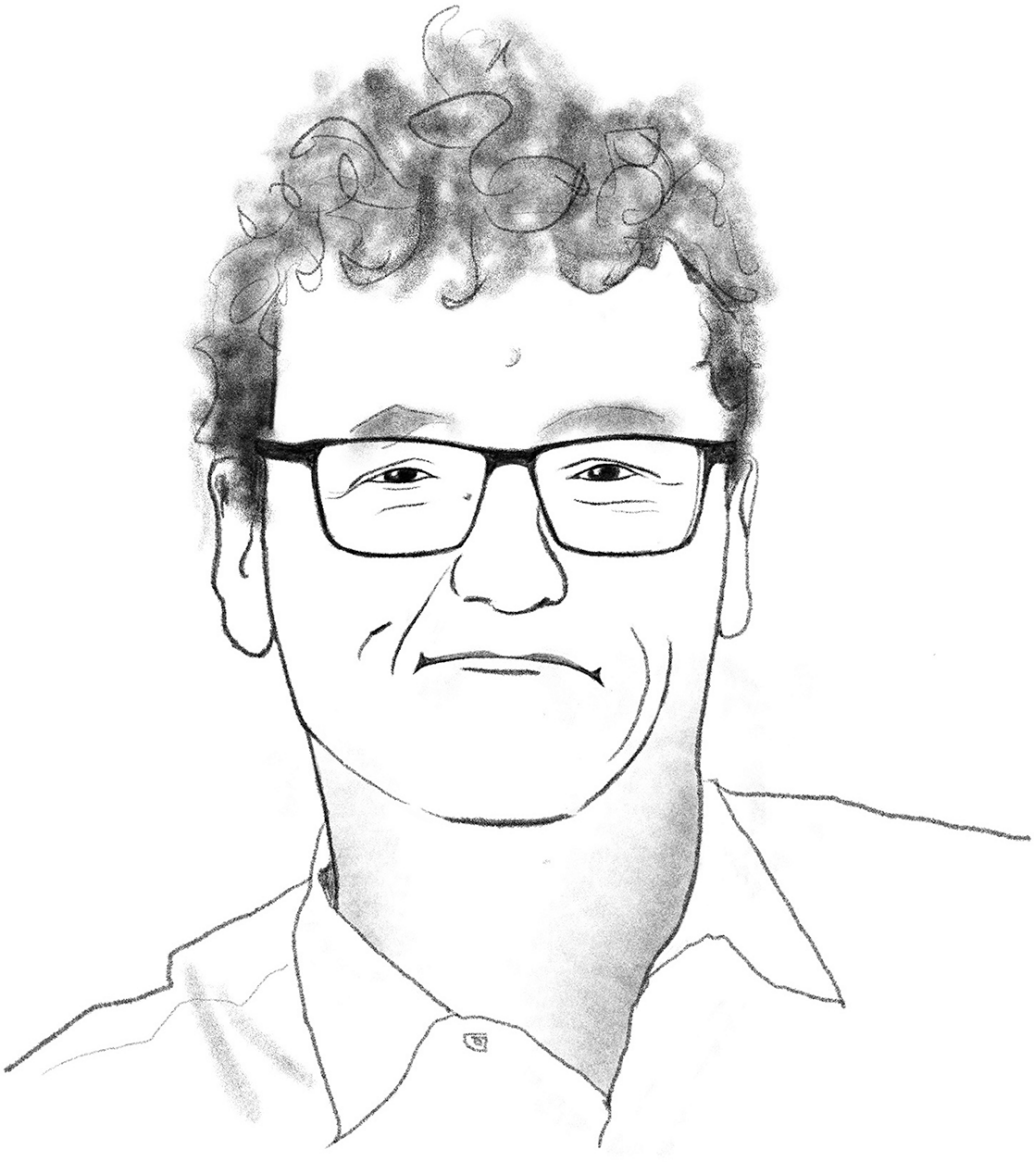

“Hundreds of organic compounds were on the shelf, and then the questions came to me, ‘Why metals? Why not organic compounds? Shouldn’t the world be developing organic catalysts that would apply to hundreds of different reactions?’ Once you come up with the right question, it becomes really a magnetic north in your mind’s compass.”

We now jump 18 months later. A question about reductive amination was raised by Tristan Lambert, later to become a distinguished academic at Columbia and Cornell, at a MacMillan group meeting. A classic Eureka moment struck MacMillan.

**Figure 3:**
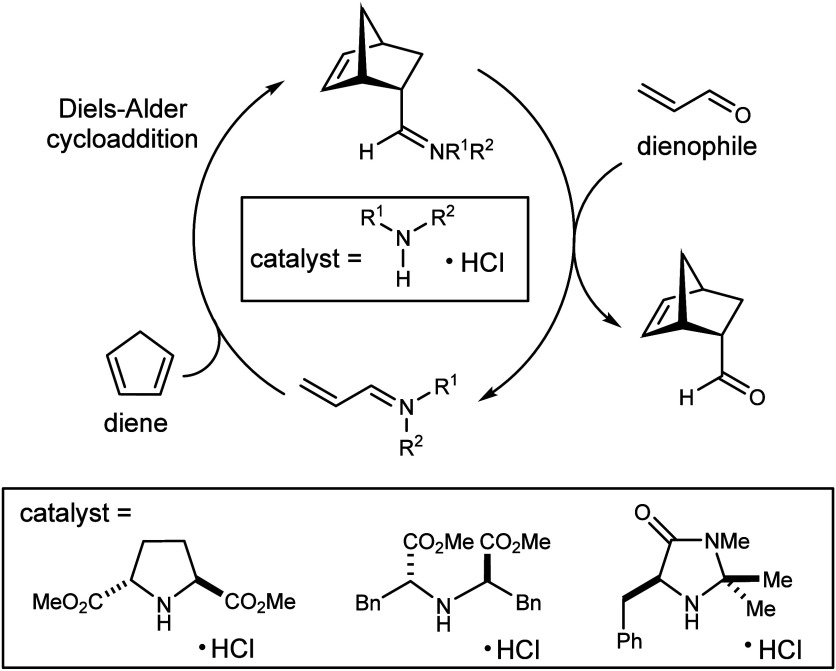
“The first highly enantioselective organocatalytic Diels-Alder reaction” by MacMillan et al. in 2000.^51^ Adapted with permission from ref (51). Copyright 2000 American Chemical Society.

“It suddenly came to me, wow, the concept of forming an iminium ion that could activate many reactions. That afternoon, we tried it and it worked [Figure 3].^51,52^

“The emotion I experienced at that moment was a combination of shock and excitement as in ‘Holy crap, I think this will work,’ along with the excitement that this could be really big, and a bit of confusion too, as in ‘It looks so simple, why has no one tried a catalytic iminium before?’

“For the record, I can still see and feel that moment in my mind, and it will stay with me forever. Tristan Lambert subsequently went back and found the notebook page where he was trying a reductive amination reaction which led to the fateful mechanism question.”

And the rest is history. MacMillan needed to have that question in the back of his head.^53^

### Herbert Mayr: Start somewhere reasonable and observe carefully

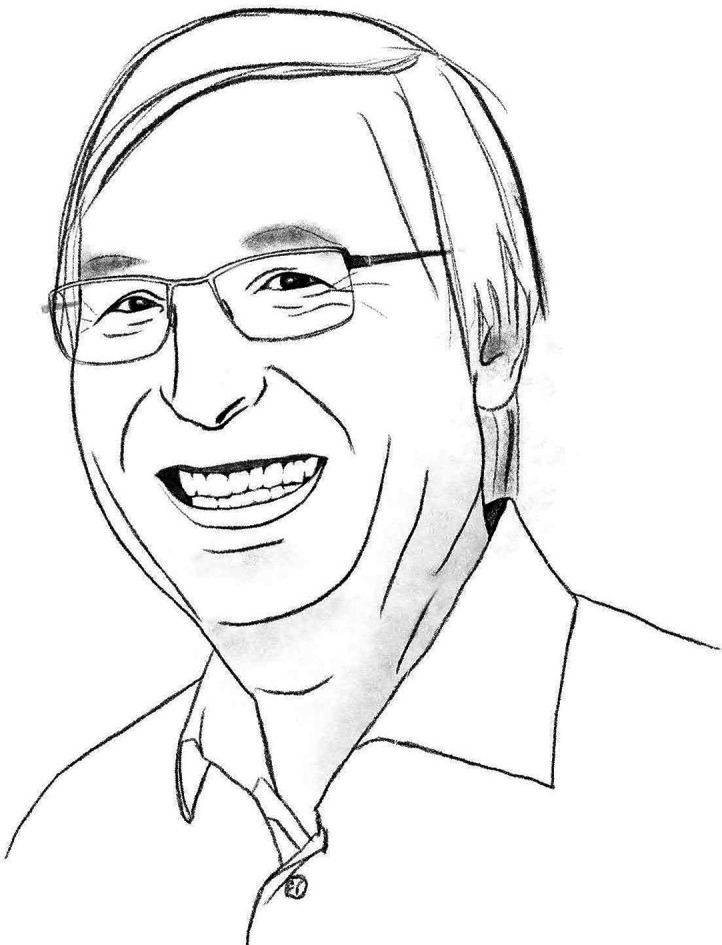



Around the time that Herbert Mayr finished his Ph.D. research with Rolf Huisgen in Munich in 1974, George Olah had just invented a way to stabilize carbocations. It was an exciting time. Mayr remembers,

“Since I knew something about cycloadditions, I went to study with George Olah at Case Western University, to learn about carbocations. I thought that, well, for my independent career, cycloadditions of carbocations might be a rewarding topic.

“This was followed by months of frustration. I initially thought that I should react a non-stabilized carbocation with an electron-rich olefin to get to a stabilized carbocation. But this gave me nothing but black tar for nine months. It was really frustrating. My concept was completely wrong.”

In desperation, Mayr tried an experiment that H. Martin R. Hoffmann had reported earlier, using silver trifluoroacetate as an initiator of carbocations. Hoffmann did his experiments with allyl cations, Mayr did his with propargyl cations.

“This analogous experiment was not a very creative idea. But I observed an 18% yield of a side product derived from a cyclic vinyl cation,^54^ which had been considered to be a terribly unstable species.”

Mayr thought to himself, “How is this possible? How did I make a carbocation which is really unstable?” Thinking about how this could happen pointed him toward the right direction: The conversion of π- into σ-bonds provided the thermodynamic driving force.

“I realized that I have to start with a stabilized carbocation and get to a less stabilized carbocation, which is trapped irreversibly, and this was the breakthrough.”^55^

Using this principle, he developed not only new synthetic methods via carbocations but also a straightforward method to measure the kinetics of the reactions of carbocations with alkenes.^56^ The observed selectivity triggered the development of Mayr’s comprehensive nucleophilicity scales, which presently compare more than 1200 nucleophiles of unprecedented structural variety and provide a unique ordering principle of organic reactivity, most useful for designing novel syntheses.^57^

Can one repeat such experiences? Do we search for ideas or do they come to us? According to Mayr,

“Some scientists start with great ideas from the beginning. However, as I have described, the concept I had initially was completely wrong. And so, I think reviewers should not be too critical with proposals of young scientists. Even when moderately exciting work is done carefully, nature often makes suggestions, which can lead to something new. It is important to observe carefully, and then try to explain why things go this way or that. And this process is, in my view, critical and may yield more important results than proposals claiming to solve the most urgent problems of our society.”

### Saundra Yancy McGuire: Applying to teaching what you have learned about yourself and your own learning processes

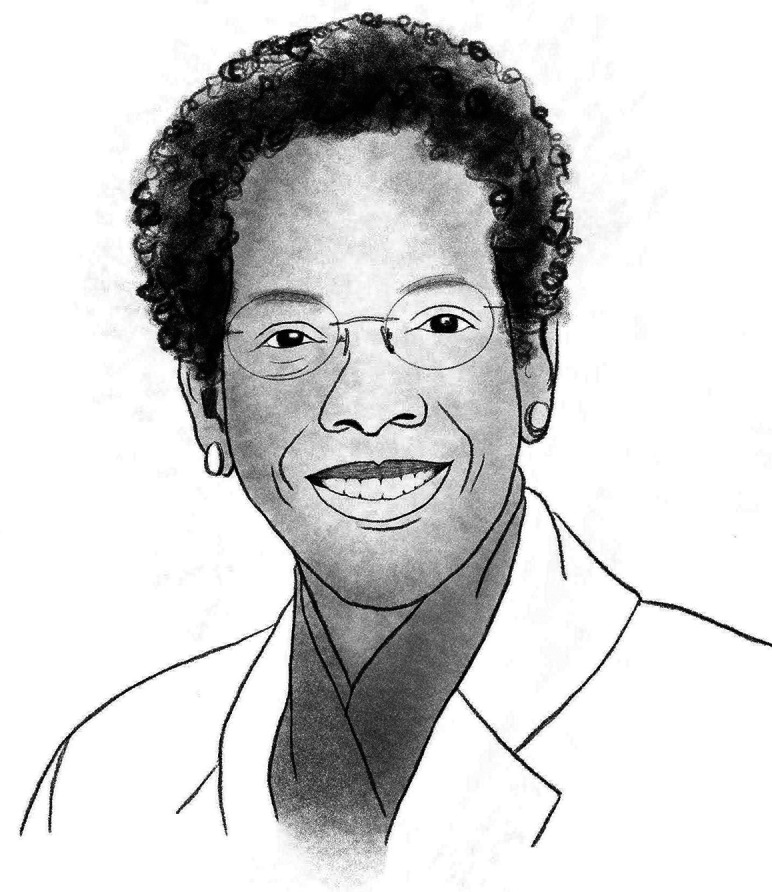



Saundra Yancy McGuire entered graduate school at Cornell in the fall of 1970 with many misconceptions.

“I thought it was only the sharper, brighter students who could excel in science courses. As for the other students, I thought they had to accept their fate to pursue less academically challenging careers. Because I had done very well as an undergraduate without putting in much study time, I thought of myself as one of the brighter students. I entered Cornell with optimism.

“Although I went to all my classes and studied really hard the night before each exam, just as I had done as an undergraduate, I did not do well, ending the semester with a low B in each class. I now know I had been operating at the lowest level of learning—in memorization mode—and it did not serve me well.

“I also had my very first experience as a teaching assistant that fall. When I entered the classroom for the very first time, I was confident I was prepared to teach the class. But in the middle of that first session, I realized that I did not fully understand what I was trying to teach. It was terrifying. Immediately I understood that I had to know the subject inside and out, at a level even deeper than the level at which I had to teach it.

“I then experienced a moment of great pedagogical growth. I recognized that I needed to apply what I had just learned about myself to my own students. Just as I needed to master the subject by discovering its underlying principles and understanding its conceptual structure, so did my students. During that first semester, I came to believe that anyone could master chemistry provided they had the desire and were shown how to concentrate on the *whys*, *hows*, and *what ifs* rather than just the *whats.*

“As soon as I put these ideas into practice, I began to see students deepen their learning and improve their grades. I discovered that mastering chemistry was available not just to the so-called “smart” students, but to all students. I felt like a physician who had discovered a life-saving treatment! I could actually help students who had lost all hope because chemistry seemed like an insurmountable obstacle to their career goals. I also began to realize that whether students are successful depends a lot on whether someone shows them how to think and learn more deeply and reflectively.”

In that moment back in 1970, Saundra Yancy McGuire’s life had changed. A period of self-reflection had improved not only McGuire’s life but, ultimately, the lives of the many faculty and students who have profited from her publications on metacognition.^58,59^ Her paradigm-shifting method for teaching students and teaching teachers^60^ how to learn has won her many awards including the Presidential Award for Excellence in Science, Mathematics, and Engineering Mentoring in a White House Oval Office ceremony.

### Valeria Molinero: Sensing an opportunity in the literature


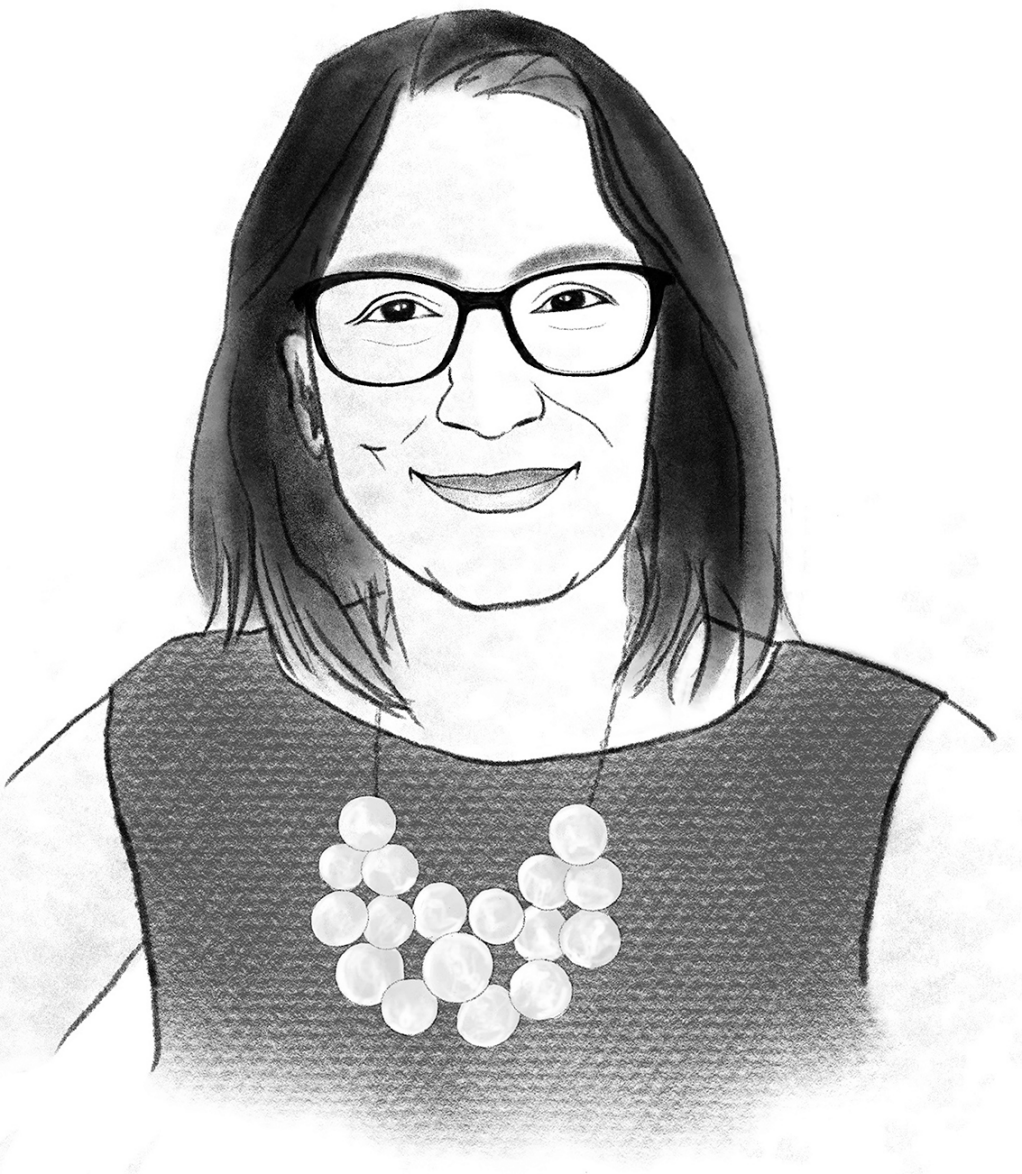
Molinero shared a memory from several years ago.

“There was a lot of buzz in the news about a paper in *Science*, an interesting publication entitled ‘Medium-density amorphous ice.’^61^ It was about a new phase of water, a topic about which I knew a lot about. But the results in this publication were surprising, and I was trying to make sense out of it. I was disturbed: the data did not fit our understanding of water. I kept asking myself, ‘What was going on?’

“It was snowing here in Utah. It was 4 am, maybe 5 am in the morning. I finally got it! My husband asked, ‘Why were you up all night?’ I tried to explain it to him. He’s a chemist, too. But our disciplines, they are so far apart. And then I had a thought. We went outside, I took some snow, compressed it in my hands, trying to illustrate for him what was the medium density form of amorphous ice. I was so excited. Understanding for the first time, it was intoxicating.”

Molinero’s research in the field continues to this day,^62^ as does her excitement in publishing her latest results.^63^

### Larry E. Overman: Reversing course in midstream

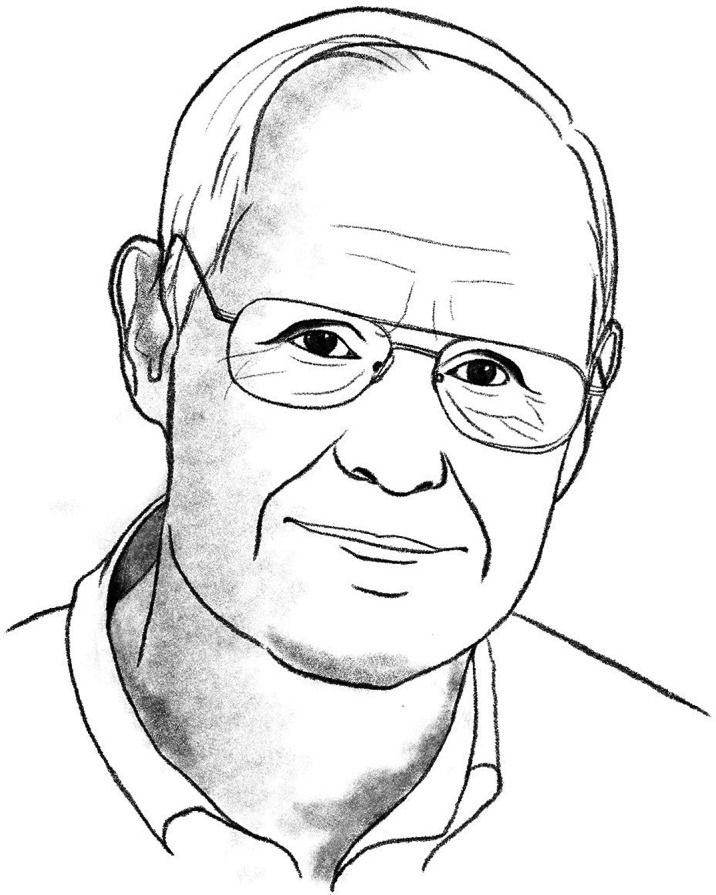



**Figure 4:**
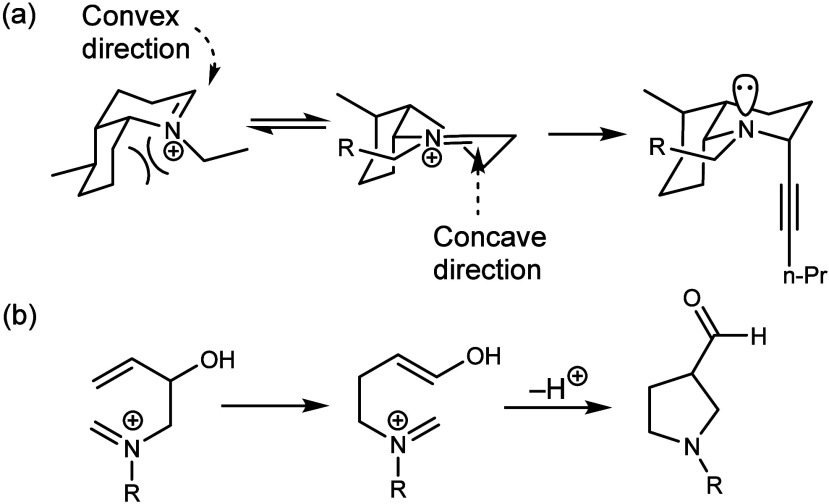
(a) Overman’s synthetic challenge and chemical insight that led to this aza-Cope-Mannich rearrangement. Adapted with permission from ref (65). Copyright 2009 Elsevier. (b) The aza-Cope-Mannich rearrangement employed by Overman in his synthesis of the perhydrogephyrotoxin.^64,65^

For Larry E. Overman, it was obtaining the wrong stereochemistry in a key step in his synthesis of the perhydrogephyrotoxin, a derivative of the skin alkaloids from poison-dart frogs of the genus *Dendrobates.*(64,65) According to Overman,

“I expected nucleophilic attack to occur from the less sterically hindered convex side of our substrate but instead we obtained the product from concave addition (Figure 4a). I was forced to think of alternative reactions, and that led me to think, if we did an iminium ion rearrangement, we might get the opposite stereochemistry. This led us to the aza-Cope-Mannich reaction (Figure 4b) which ultimately was enhanced by appropriate substitution patterns.”

This reaction is one of most powerful tools in the preparation of pyrrolidines, a common moiety in natural products chemistry. And it helped craft, indeed propel, Overman’s career. As for his emotional response to his discovery, Overman recalled,

“I had no great emotional response the moment I imagined the aza-Cope Mannich reaction or after its first successful experimental manifestation, as it took me several years to appreciate the wide applicability of this reaction.”

### Richmond Sarpong: Adopting a research strategy which incorporates a high-risk unknown, very close to the end

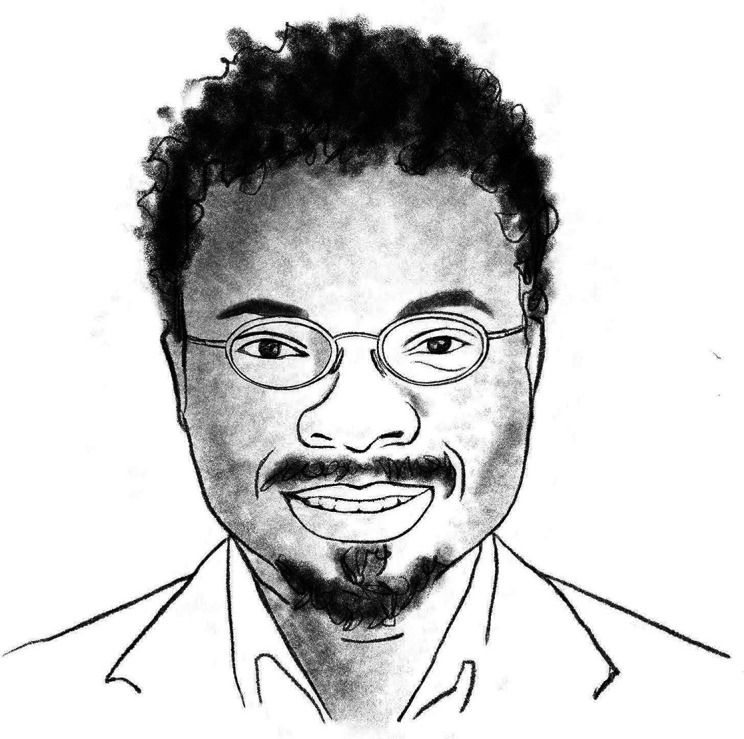



Curiosity certainly drives discovery.^66^ But often, necessity drives curiosity which then drives discovery.

**Figure 5:**
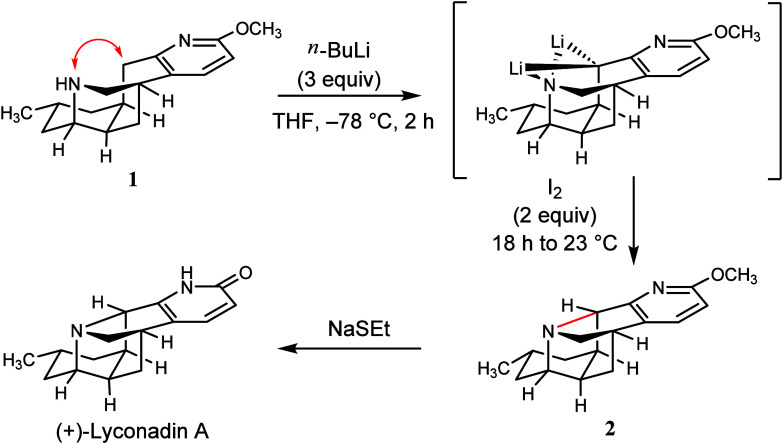
Key C–N bonding forming cyclization step in Sarpong’s total synthesis of alkaloid (+)-lyconadin A is shown by the red double-headed arrow in **1** and the new bond formed in **2** (and shown in red).^67,68^

It was necessity and ultimately desperation that drove Richmond Sarpong and his students to develop a novel, “highly efficient oxidative C–N bond forming reaction that relies on the union of a nitrogen anion and a carbon anion.”^67^ Perhaps foolishly, certainly optimistically, in the late 2000s, Sarpong et al. attacked the synthesis of “the architecturally complex *Lyropodium* alkaloid (+)-lyconadin A” with a dubious but compelling strategy (Figure 5). They undertook this total synthesis while knowingly lacking a method to conduct the key and near final step without. And that was a reaction without any experimental precedent.

But perhaps a “must accomplish task with no established precedent” is the perfect strategy to propel creativity and innovation. Analogously, Whitesides suggested “Go where there is no crowd”^68^ as one of the tools to stimulate curiosity. “No crowd” is like “no method.”

In this instance, the challenge is easy to describe. How to convert **1** to **2**? According to Sarpong,

“It was a slowly developing Eureka moment. We were at the final stages of the total synthesis, and we were taking a major risk. We tried many things, all aligned with making a nitrogen–carbon bond. We tried all sorts of nucleophilic and electrophilic approaches. None worked. I wasn’t in a panic. I was concerned. I was forced to think outside the box because all our standard ideas and hopes were fruitless. To turn the problem around, I flipped the chemistry around. Perhaps we could take two nucleophiles and have them come together by some type of oxidative coupling. This idea relied on some fundamental knowledge that I already had. I remembered, in Leo Paquette’s synthesis of dodecahedrane, that they used Pd/C to extract two hydrogen atoms and obtained a C–C coupling.^69^ I wondered, ‘Could we do a C–N version of that transformation?’. In hindsight, I was counting on the proximity of the carbon and nitrogen atoms in the key precursor to help. Yes, this approach was borne out of desperation, almost a throwaway idea. Before, our creativity was based on established precedents. And we succeeded!^67,68^ Later, we examined this approach—the generation and reaction of dianions—more systematically.”^70,71^

Recalling the moment, he learned that the C–N bond forming cyclization succeeded, Sarpong said, “It was a mix of amazement, pure joy, and relief! My tenure depended on that molecule coming together!”

What a gamble!

Maybe not. Perhaps the best strategy for innovation, even for tenure at a university like Berkeley, is to choose problems where seemingly impossible challenges are imbedded. Challenges that we recognize up front will require something extraordinary. And would not it be great if those challenges could be solved simply and even with some admirable degree of elegance?

Ultimately, Dave MacMillan^72^ used photoredox catalysis to achieve C–N coupling reactions well beyond Sarpong’s successes. Sarpong mused, “Maybe we missed the boat by not continuing this research.”

Or maybe the best success of one’s ideas lies in its portability,^73,74^ its usefulness, and its extendibility in the hands of others.

### Peter Schreiner: Asking almost childlike questions helps you break out of the box

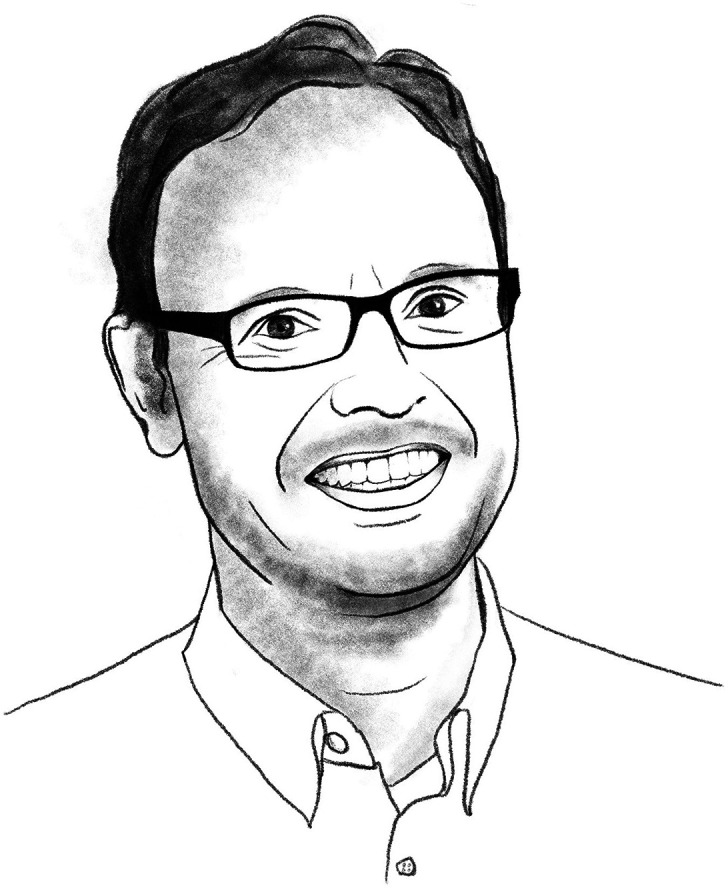



For Peter Schreiner,

“It’s being a little naïve and asking very simple questions.”

Schreiner remembers writing his first proposal as an assistant professor. He asked himself a question that perhaps all of us ask frequently, “What can I do that is truly new?” While reading about metal-catalyzed reactions, Schreiner mused, half-jokingly,

“What if we leave out the metal? Could catalytic activity be retained through other types of molecular interactions?”

**Scheme 1:**
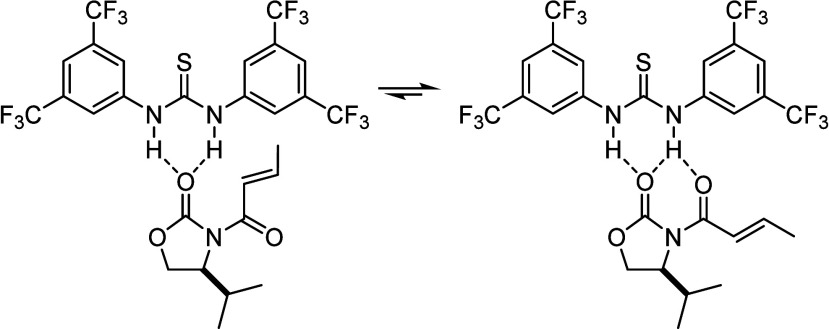
*N,N′*-Bis[3,5-bis(trifluoromethyl)phenylthiourea, also Known As Schreiner’s Thiourea Catalyst, Acting Like a Lewis Acid Catalyst

Starting from an amine base, Schreiner designed a thiourea catalyst (Scheme 1), and shortly thereafter the story of hydrogen bond catalysis unfolded.^75^

“Being exposed to a lot of different research and wandering between theoretical and experimental chemistry helps you ask questions from different angles. It’s like traveling to a different country. When you are there, you see what is good and what is bad about your own country, and you see things from a new perspective....It gets harder to retain the child inside. But it’s certainly possible to replicate these moments.”

### Lawrence T. Scott: It is listening very carefully

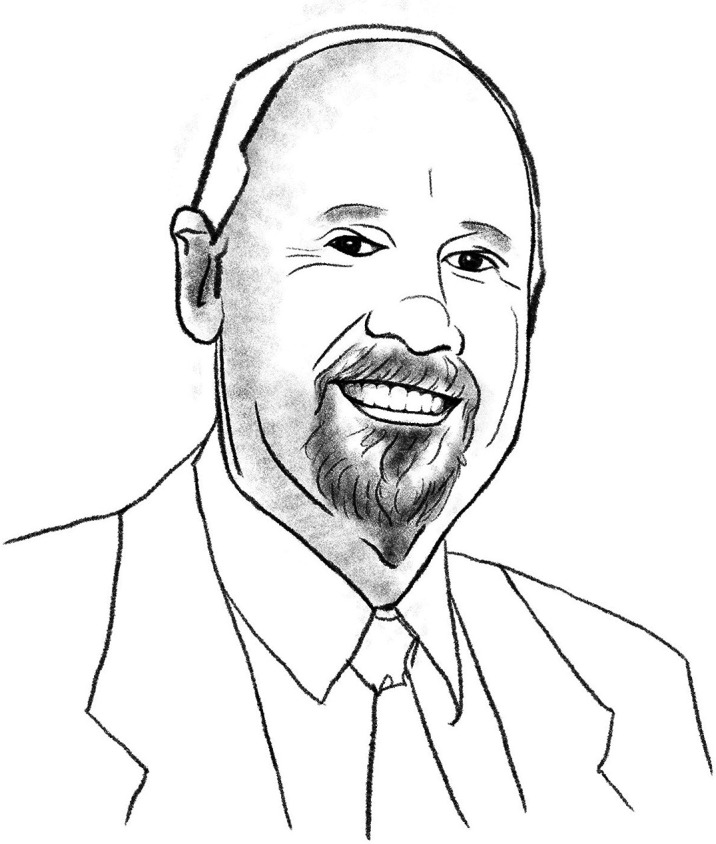



For Larry Scott, “It’s interacting with others in a fruitful environment.”

Scott remembers hearing Harry W. Kroto’s talk at the 1989 ISNA (International Symposium of Novel Aromatic Compounds) meeting in Osaka, Japan. “C_60_ has also been detected in flames,” reported Kroto.

Scott was astounded by Kroto’s comment and immediately realized the opportunity in front of him. He recalled thinking, “Flash Vacuum Pyrolysis (FVP) could be the secret to synthesizing C_60_ in the lab!” Scott reasoned that FVP triggers high-temperature chemistry in the gas phase, just like in flames, but without oxygen. He also knew that intramolecular aryl–aryl coupling reactions (cyclodehydrogenations) were well-known under FVP conditions, and the high temperature would supply the energy needed to bend the molecules in the direction needed for the desired structure. By the late 1980s, Scott was the world’s expert in high temperature pyrolysis of aromatic and nonbenzenoid aromatic compounds.^76−79^

**Figure 6:**
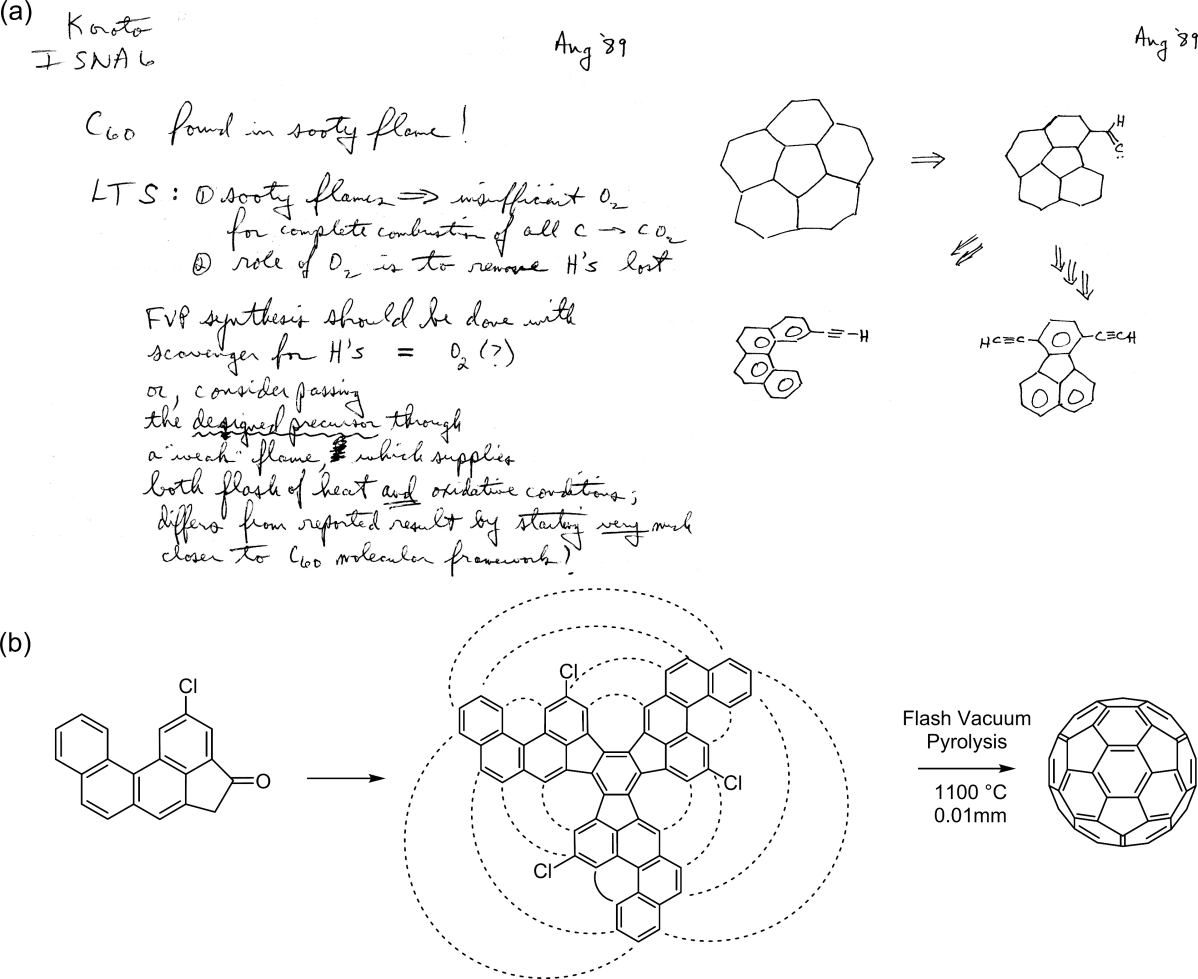
(a) Excerpts from Larry Scott’s notebook from August 1989 on the synthesis of corannulene. (b) An outline of key steps of Scott’s rational synthesis of C_60_ reported in 2002.^82^

On the flight home from Japan, Scott filled his notebook with pages and pages of possible ways to stitch together planar precursors into the ball-shaped molecule. His subsequent FVP synthesis of corannulene (Figure 6a)^80^ provided the first proof of the principle that bowl-shaped polyarenes could be synthesized from planar hydrocarbon precursors by FVP. Extending the method to larger and larger geodesic polyarenes^81^ provided invaluable lessons about the scope and limitations of FVP as a synthetic method. Kroto’s comment inspired an “Aha!” moment. From there, it was just a matter of finding the right precursor and testing Scott’s hypothesis. Ultimately, one of those original sketches successfully led to the first rational synthesis of C_60_ in isolable quantities (Figure 6b).^82,83^

### JoAnne Stubbe: Literature results pointed the way

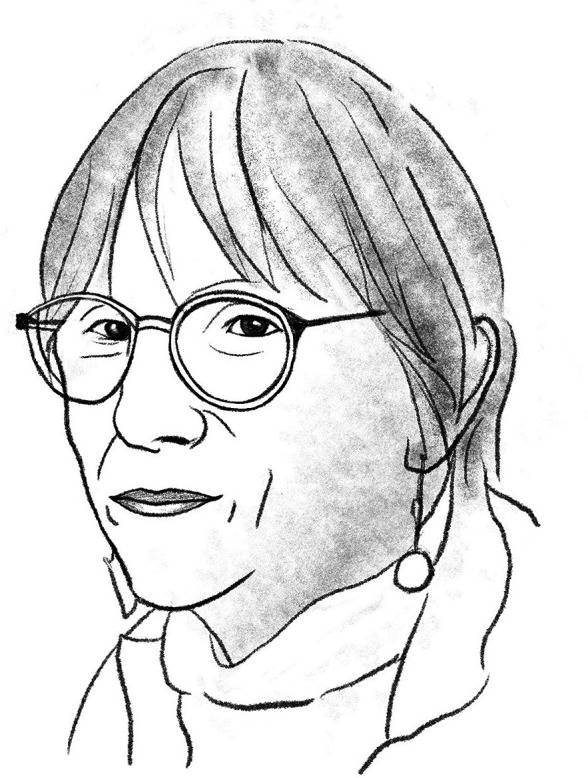



Over 40 years ago, early in her career, JoAnne Stubbe began her research on ribonucleoside-diphosphate reductase (RDPR). This enzyme supplies 2′-deoxynucleotides required for DNA synthesis and is thus essential for cell growth.

**Scheme 2:**
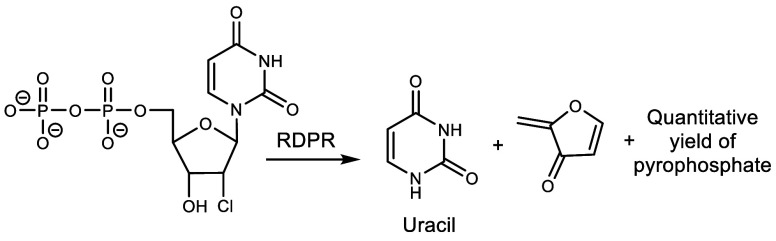
Inactivation of Ribonucleoside-Diphosphate Reductase (RDPR) by Its Reaction With 2′Chloro2′Deoxyuridine5′-Diphosphate

In the late 1970s, Stubbe was studying the inactivation of this enzyme by its reaction with 2′-chloro-2′-deoxyuridine 5′-diphosphate (Scheme 2).

The initial goals were to determine the reaction products of this reaction, then its mechanism, which Stubbe achieved.^84−86^ As Stubbe recalled a particular innovative and life-changing moment,

“I was then in the Pharmacology Department at Yale. We did not have a good NMR instrument, so I was running the NMR late at night at Yale’s Department of Chemistry. I took a phosphorus NMR spectrum of the product, and I saw a singlet. It was spectacularly thrilling. Thrilling. In my mind, I concluded, ‘Pyrophosphate!’ We had several ideas as to the mechanism of the reaction, but I did not know we’d see pyrophosphate.

“As with most of our discoveries, I experienced an amazing high!!”

That moment, that reaction, led to many research projects and helped propel Stubbe to her 2009 National Medal of Science awarded by President Obama and her 2020 Priestley Medal of the American Chemical Society.

### Myunghyun Paik Suh (Seoul National University): It came so easily. It was so simple

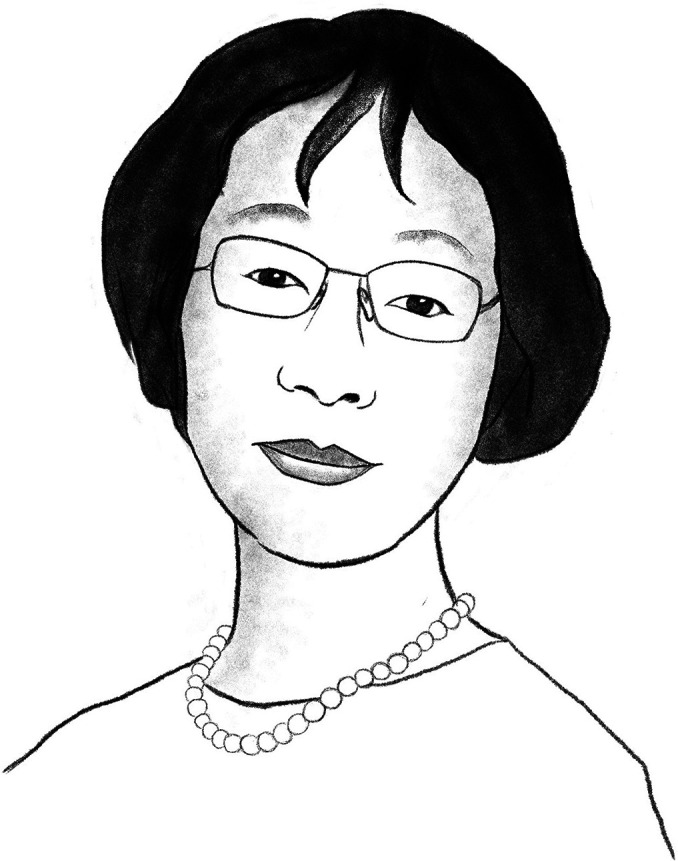



Breakthrough science is of two kinds: Those which colleagues immediately accept but happily look for exceptions, if not violations, to the new order. And those which colleagues refuse to believe at the outset, saying things like, “This must be false, no one has ever seen anything like that before.”

Myunghyun Paik Suh’s observation in 2000 was of the second kind. She believed that she had observed a single crystal to single crystal transformation. She reported “a metal-organic bilayer open framework which retained its single crystallinity upon removal and exchange of guest molecules during redox reactions”^87−89^ in a submission to *Nature*. But one of the reviewers did not believe Suh’s claim. This reviewer proposed the dissolution of the crystal followed by re-nucleation on the crystal surface for the guest exchange and oxidation reactions, rather than the single-crystal to single-crystal transformation. The submission was rejected.

This was certainly neither the first time^90^ nor the last time a novel scientific observation would be ostracized by a belief system based on interpolative rather than extrapolative thinking. It is a real paradox within science: to turn away exactly what is or should be most valued: novelty and uniqueness.

**Figure 7:**
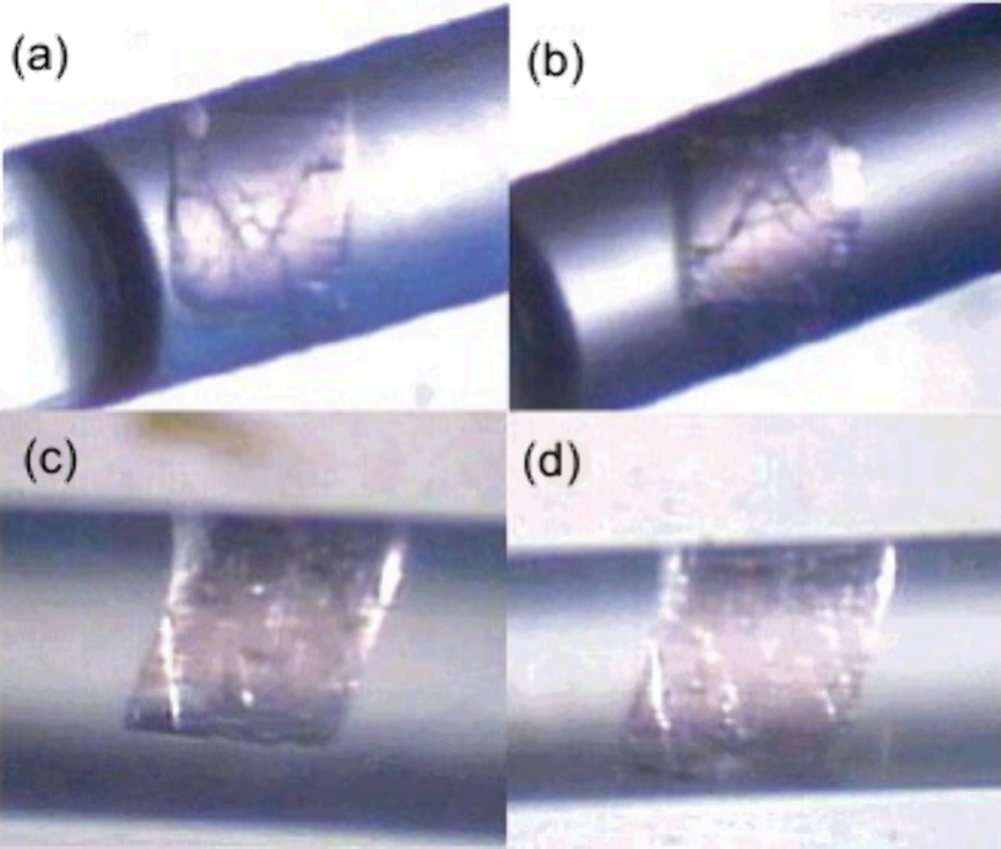
Photographs of Suh’s crystal in guest-exchange processes.^87,88^ (a) The initial single crystal of [Ni_2_(C_26_H_52_N_10_)]_3_[BTC]_4_·6C_5_H_5_N·36H_2_O (**1**), where BTC = 1,3,5-benzenetricarboxylate, was immersed in pyridine within a glass capillary. The photograph was taken immediately. (b) After immersion of **1** in pyridine for 24 h. No change was noted, but [Ni_2_(C_26_H_52_N_10_)]_3_[BTC]_4_·20C_5_H_5_N·6H_2_O was formed. (c) This photograph was taken as soon as **1** was immersed in benzene within a glass capillary. (d) After immersion of **1** in benzene for 24 h, [Ni_2_(C_26_H_52_N_10_)]_3_[BTC]_4_·14C_6_H_6_·19H_2_O was formed. Reprinted (adapted) with permission from ref (88). Copyright 2004 American Chemical Society.

Suh was in her office on the evening she received *Nature*’s rejection. She was contemplating what to do next. There was no suggestion of “submit after revision.” And then, rather spontaneously, the idea came to her. Take photographs under an optical microscope to establish the retention of the crystal’s single crystallinity “during and after immersion of the crystal in the solvent as well as during the redox reaction in the solution.”^87,88^ This was done, and no change was seen in size, morphology, and transparency (Figure 7). Suh subsequently submitted an enhanced, indeed much stronger, manuscript containing these new results to the *Journal of the American Chemical Society.* One of the reviewers of Suh’s submission wrote,

“This paper is substantially improved over an earlier version I reviewed for *Nature*....I am fairly convinced now that these reactions are true guest exchange rather than simply renucleation at the surface and growth of a new phase.”

And thus, the manuscript was published in *JACS*, and a new branch of materials science was initiated.^91,92^

## Discussion of Results

In his 2007 publication entitled “The Cha-Cha-Cha Theory of Scientific Discovery,” Koshland pointed out that there are three types of discovery: chance (or serendipity), charge, and challenge.^7^ “Chance” is “the faculty of making happy and unexpected discoveries by accident.”^16^ “Charge” refers to solving specific and obvious problems, often dealing with a societal need. “Challenge” refers to solving long-standing scientific puzzles or incongruities. As discussed in the Introduction, we posit that Eureka moments can occur during all the stages of discovery. There surely is a fourth “C”, namely “Curiosity,”^66,93^ often cited in the above interviews.

The stories collected here make a case for retrospective studies of Eureka moments. Our study is an interview-intensive examination of a small number of individuals rather than a statistically based research program involving a large number of respondents answering focused though less nuanced questions. We believe that at least two characteristics are shared by all our interviewees. First, each interviewee reported an awareness of a discontinuity in their knowledge, from unknowing to knowing. Second, once connections were made by our interviewees and “the light bulbs were turned on,” the research goals became clearer, and the struggles were then a more straightforward challenge. Those who experience the Eureka moment and make the leap from the discontinuity in knowledge are innovators, whereas those who do not recognize or cannot take advantage of the discontinuity do not make the discovery. But even among those who recognize the discontinuity in knowledge, there can be a substantial barrier to finding the right solution. Nonetheless, our interviewees focused more on the intellectual—even spiritual—Eureka moments rather than details of their entire discovery and solution processes.

Of course, the history of chemistry is not without its examples of Eureka moments.^12^ The most famous of all is Friedrich August Kekulé’s dream of the structure of benzene, which may or may not have actually occurred as self-reported.^94−103^ The trouble is, there are just too few case studies on events like Kekulé’s,^104^ and to our knowledge, none in chemistry that are broad in scope and that involve multiple interviewees.

As shown in Table 1, Novik and Sherman^105^ and Sprugnoli et al.^106^ divided the classes of problem solving into three types. We have placed each of our 18 cases within these three general categories. There is wide representation within each of the three types. Table 1 only hints at the great variability among the individual interviewee’s experiences. Each case has its very own set of periods of preparation, incubation, illumination and recognition, and consciousness, the latter including definition, verification, appreciation, and continuation.^19^ And each case has its own type of emotional response.

**Table 1:** Placement of Each of our 18 Interviewees within Novick and Sherman’s and Sprugnoli et al.’s Three-Factor Eureka Moment Organization.^a^^,^^105,106^

characteristic		examples from interviews conducted herein
**1**	Analytical problem-solving which can be reconstructed into a series of logically oriented steps that can be identified;^106^ search^105^	Alabugin (in the library)
		Clemons (trial-and-error, prepared new crystals, X-ray crystallographic analysis) (Charge^b^)
		Hariharan (trial-and-error, crystal engineering and chromophore design)
		Kauzlarich (trial-and-error, synthetic inorganic chemistry)
		Overman (while thinking about alternative reaction strategies)
		Sarpong (thinking outside the box)
		Suh (solving an immediate logical roadblock)
**2**	A memory retrieval process of previously acquired knowledge^105,106^	Hoffmann (during standard research trajectory, accumulated theoretical chemical data, more calculations)
		McGuire (while teaching) (Challenge^b^)
		Scott (inspired by a lecture, thinking about the literature)
		Stubbe (an experimental result forced a conclusion)
**3**	Insight characterized by a sudden and unexpected understanding, “pop-out”,^105^ sometimes serendipitous	
**3a**	While searching for a solution	Freedman (on mindful “innovation walks”)
		MacMillan (while doing other experiments)
		Mayr (while planning his future research program)
**3b**	While searching for a problem to solve	Francisco (designed quiet time, at home)
		Schreiner (asked, what could he do that is truly new?)
**3c**	When not searching at all (sometimes by Chance^b^)	Langer (on an exercise bicycle in a hotel)
		Molinero (while reading the literature)

a Note that there is wide diversity within each of these three categories and that the third categories has three identifiable subsets, not specified by Novick and Sherman^105^ or Sprugnoli et al.^106^

b “Charge, challenge, and chance” refer to Koshland’s three categories of discovery^7^ discussed above.

Table 1 also includes some overall similarities. Each of these categories likely is preceded by a period of confusion followed by a Eureka moment and a period of delight followed by research consequences.^107^ Almost all entries in Table 1 illustrated thinking “outside the box.”^108−110^ Can a “trial-and-error” approach also be “thinking outside the box?” We believe it can and very much so, e.g., when the various “trials” involve unconventional, eccentric, even maverick or counterculture ideas. All these involve outside-the-box thinking, and a struggle to do that.^111^ Being an outsider to a field can help, too.^112^ The recollections provided by our interviewees exemplify Koshland’s trio admirably (see Table 1) and mirror stories and historical events about science and serendipity collected in other sources; see for example, *Science& Serendipity*(113) by Ernest L. Eliel (past president of the American Chemical Society) and *The Travels and Adventures of Serendipity* by Robert K. Merton and Elinor Barber.^114^ We wonder if every discovery encompasses elements of serendipity as well as targeted and curiosity-driven problem solving.

## On Learning

Can Eureka moments be fostered, produced by training, and/or encouraged to happen? Several of our case studies suggest “yes.” Joseph Francisco sets aside Christmas Eve for his most important ideation time. Danna Freedman goes on long walks. Lawrence Scott interacts with people and engages with a fruitful scientific community. Robert Langer is always imagining.

Most research into the learning process and Eureka moments lies within the realm of entrepreneurship education.^115−117^ This more focused literature suggests three types of “action and problem-based learning” that have been recognized as key to entrepreneurial learning,^116^ and these surely have applicability within chemistry. These are problem-based learning, experiential learning, and inquiry-based learning. The relationship between these types of learning and Table 1 is evident. Given that Eureka moments come rarely and unexpectedly, staged or experimental Eureka moments have to be generated in order to examine such occurrences experimentally. The relevance of these artificial experiments^106,107^ to real life Eureka moments is obviously open to question.

Some habits are likely important for enhancing serendipity. As proposed by Anderson, making space for spontaneous moments, encouraging playfulness, engaging with imperfect information, and seeking uncertainty can serve as imaginative motivators.^18^ Specific examples include chance conversations, engagements with idiosyncratic outliers and inconsistent data, and unreasonable or unanticipated or anomalous results.^118^ These interviews suggest that one should search for connections from totally (and seemingly) unrelated information, even casual remarks. The literature points to the need for eternal vigilance: to keep one’s eyes open for hints within one’s current research, in the literature, and in every lecture and in every discussion. One should honor serendipity.^114^ And one should dare to be bold, to imagine possibilities, and to interact with the environment.

The distinguished chemist R. B. Woodward often pointed to his Muse for stimulation and encouragement. We present several excerpts from his unpublished letters:

“I do wish I were able to help in the project outlined in your letter of February 24th. But the Muse is not upon me, and it is unlikely to be before I leave shortly for Switzerland.”^119^

and

“At present, I haven’t a clue, even in the most general sense, of what line I might pursue [for the lecture in London honoring Sir Robert Robinson] and even less of when I might be seized of the Muse....”^120^

and

“I have realized at once that what was needed was a rallying cry which, sweeping all before it, would reverse the melancholy trend. So I summonsed the Muse, and am glad to offer the accompanying modest result.”^121^

Perhaps Woodward’s Muse was related to Roald Hoffmann’s mode of inspiration:

“Sometimes asking the right question makes the solutions come.”^122^

## Conclusions

Discovery in science has many steps, each of which can provide Eureka moments.

What do these interviews and these stories tell us? For one, no one seemed hesitant to relate to their personal Eureka moments, as they were all success stories. But these experiences required each chemist to step openly and willingly into a region of discomfort, uncertainty, and ambiguity, and possible failure.^18^ But this is the milieu of scientific research.

These stories present different ways that chemists approach problem solving and ambiguity.^123^ While we strove for some generalizations (as scientists do), we must not forget about individualities and idiosyncrasies shared with us by our interviewees. As interviewers, our most striking observation is the diversity in experiences related to us by the interviewees. There are likely as many paths leading to a Eureka moment as there are scientists and research opportunities! Each in their own ways, in their timing and depth of thinking and reliance on the published literature, they are all very different from each other. We add one more observation: Most of our interviews were brief. Very brief. We posed the question, the interviewees responded directly and easily. That comfort in response speaks to the authenticity of their reports.

Navigating the path to creativity involves a multifaceted approach, and the experiences of our interviewees illuminate some shared traits. For some, it begins with careful observation, embracing unexpected findings, and consciously removing bias. For others, it is the courage to question. Creativity demands boldness, and creative chemists are not afraid to challenge conventional wisdom and to imagine untread possibilities. For others, it is interdisciplinary thinking—drawing inspiration from diverse research areas and translating questions and solutions across scientific fields. For still others, moments of creativity can come from collaboration and networking—fostering an environment where ideas can be exchanged and refined (or jump out at you in the middle of a talk!). Still others seek quiet and contemplation. Creativity requires patience, and some chemists are better prepared for a longer-term engagement. Creative chemists understand the value of stepping back, taking breaks, and allowing time for ideas to develop (or bubble up).

We marvel in the diversity of discoveries and inventions, diversity of context, and diversity of scientist’s personalities.^19^ We marvel in the differences in terms of the need to focus on a specific problem or to abandon it for a while, to study the relevant literature or not, to be respectful of the nature of one’s emotional state, agitated or relaxed. This report speaks to pluralistic experiences. What works for any one person is surely a function of personality, surroundings, resources, and opportunities. But surely windows of opportunity open for all of us.

## Coda

This publication would be incomplete without a citation to science cartoonist Sidney Harris’s most recent self-published volume *Eureka Details to Follow: Cartoons on Chemistry*.^124^ Quite appropriately, this collection of cartoons received an excellent review in *American Scientist.*(125) Sidney Harris is a genius.^126^

-----

Too bad we do not have a collection of interviews such as these, assembled every 10 or 25 years, stretching back to the days of Joseph Priestley and Antoine Lavoisier and even earlier. How much more we would understand about and appreciate the past and our own place in this continuing history of chemistry.

## Dedication

Dedicated to the memory of Albert Eschenmoser (1925–2023), a scientist who surely experienced many Eureka moments and provided Eureka moments to the admirers of his research.
